# Association of Inferior Vena Cava Filter Placement for Venous Thromboembolic Disease and a Contraindication to Anticoagulation With 30-Day Mortality

**DOI:** 10.1001/jamanetworkopen.2018.0452

**Published:** 2018-07-13

**Authors:** Tyson E. Turner, Mohammed J. Saeed, Eric Novak, David L. Brown

**Affiliations:** 1 Division of Cardiology, Department of Medicine, Washington University School of Medicine in St Louis, St Louis, Missouri; 2 Division of Infectious Diseases, Department of Medicine, Washington University School of Medicine in St Louis, St Louis, Missouri

## Abstract

**Importance:**

Despite the absence of data from randomized clinical trials, professional societies recommend inferior vena cava (IVC) filters for patients with venous thromboembolic disease (VTE) and a contraindication to anticoagulation therapy. Prior observational studies of IVC filters have suggested a mortality benefit associated with IVC filter insertion but have often failed to adjust for immortal time bias, which is the time before IVC filter insertion, during which death can only occur in the control group.

**Objective:**

To determine the association of IVC filter placement with 30-day mortality after adjustment for immortal time bias.

**Design, Setting, and Participants:**

This comparative effectiveness, retrospective cohort study used a population-based sample of hospitalized patients with VTE and a contraindication to anticoagulation using the State Inpatient Database and the State Emergency Department Database, part of the Healthcare Cost and Utilization Project of the Agency for Healthcare Research and Quality, from hospitals in California (January 1, 2005, to December 31, 2011), Florida (January 1, 2005, to December 31, 2013), and New York (January 1, 2005, to December 31, 2012). Data analysis was conducted from September 15, 2015, to March 14, 2018.

**Exposure:**

Inferior vena cava filter placement.

**Main Outcomes and Measures:**

Multivariable Cox proportional hazard models were constructed with IVC filters as a time-dependent variable that adjusts for immortal time bias. The Cox model was further adjusted using the propensity score as an adjustment variable.

**Results:**

Of 126 030 patients with VTE, 61 281 (48.6%) were male and the mean (SD) age was 66.9 (16.6) years. In this cohort, 45 771 (36.3%) were treated with an IVC filter, whereas 80 259 (63.7%) did not receive a filter. In the Cox model with IVC filter status analyzed as a time-dependent variable to account for immortal time bias, IVC filter placement was associated with a significantly increased hazard ratio of 30-day mortality (1.18; 95% CI, 1.13-1.22; *P* < .001). When the propensity score was included in the Cox model, IVC filter placement remained associated with an increased hazard ratio of 30-day mortality (1.18; 95% CI, 1.13-1.22; *P* < .001).

**Conclusions and Relevance:**

After adjustment for immortal time bias, IVC filter placement was associated with increased 30-day mortality in patients with VTE and a contraindication to anticoagulation. Randomized clinical trials are needed to determine the efficacy of IVC filter placement in patients with VTE and a contraindication to anticoagulation.

## Key Points

**Question:**

What is the association of inferior vena cava filter placement with 30-day mortality in patients with venous thromboembolic disease and a contraindication to anticoagulation?

**Findings:**

In this cohort study, using 2 different statistical methods with adjustment for immortal time bias, inferior vena cava filter placement in patients with venous thromboembolic disease and a contraindication to anticoagulation was associated with an increased risk of 30-day mortality.

**Meaning:**

Randomized clinical trials are needed to define the role of inferior vena cava filter placement in patients with venous thromboembolic disease and a contraindication to anticoagulation.

## Teaser

This comparative effectiveness cohort study explores the association of inferior vena cava filter placement with 30-day mortality in patients with venous thromboembolic disease and a contraindication to anticoagulation.

## Introduction

Venous thromboembolism (VTE), which includes both pulmonary embolism (PE) and deep vein thrombosis (DVT), is a significant cause of morbidity and mortality worldwide, with an incidence of 117 cases per 100 000 person-years^1^ and a 1-year mortality for PE of at least 22% in patients with Medicare.^2^ Treatment of patients with VTE is based on anticoagulation, but for many patients, this therapy is contraindicated owing to recent surgery or underlying coagulopathies. In patients with VTE and a contraindication to anticoagulation, major professional societies, including the American College of Chest Physicians,^3^ American Heart Association,^4^ Society of Interventional Radiology,^5,6^ American College of Radiology,^7^ and the British Committee for Standards in Haematology^8^ recommend consideration of IVC filter placement. While IVC filters have been widely available since the 1960s, their use in the United States has steadily and dramatically increased from 2000 procedures in 1979 to more than 100 000 procedures in 2005.^9^ More recently, rates have begun to decline with approximately 96 000 procedures in 2014.^10^ This use has occurred despite the absence of data on a mortality benefit associated with IVC filter placement. An early randomized clinical trial showed a reduction in the number of symptomatic PEs but no reduction in mortality after IVC filter insertion. Importantly, this trial excluded patients with a contraindication to anticoagulant therapy,^11^ which is, to our knowledge, the most widely accepted indication for IVC filter placement and the only indication for which the several professional societies agree. Retrievable IVC filters were analyzed in a randomized clinical trial^12^ of patients with PE and a high likelihood of recurrence. In this trial,^12^ IVC filters did not lower recurrent PE, but patients with a contraindication to anticoagulation were excluded. Thus, the findings of existing randomized clinical trials are not applicable to a large segment of the VTE population.

As a result, in the last 3 years, observational studies have attempted to understand the association between IVC filter placement in patients with VTE and a contraindication to anticoagulation and their outcomes. These studies have often failed to adjust for immortal time bias, which is the interval between hospital admission and IVC filter placement, during which time death cannot occur in the intervention group but can occur in the control group.^13^ Failing to account for this potential source of bias can erroneously skew the results in favor of the intervention by falsely conferring a survival advantage to the treated group.^13^ Given this concern for unaccounted biases in the context of a relatively common invasive procedure for which there is no evidence of a mortality benefit, we conducted an analysis incorporating adjustment for immortal time bias using 2 different statistical models to evaluate the outcomes of IVC filter placement in patients with VTE and a contraindication to anticoagulation.

## Methods

This comparative effectiveness, retrospective cohort study used the State Inpatient Database (SID) and the State Emergency Department Database, a part of the Healthcare Cost and Utilization Project of the Agency for Healthcare Research and Quality, from hospitals in California (January 1, 2005, to December 31, 2011), Florida (January 1, 2005, to December 31, 2013), and New York (January 1, 2005, to December 31, 2012).^14,15^ This study conformed to the International Society for Pharmacoeconomics and Outcomes Research (ISPOR) reporting guideline. The SID includes inpatient discharge records from nonfederal, short-term general hospitals. The SID data sets from California, Florida, and New York contain an encrypted person identifier allowing longitudinal follow-up. Records with a missing person identifier and records from psychiatric, dependency, and rehabilitation hospitals were excluded. Same-day hospital transfers were considered a single hospitalization. Each SID record contains *International Classification of Diseases, Ninth Revision, Clinical Modification* (*ICD-9-CM*) diagnosis and procedure codes. Diagnosis codes are assigned at discharge without corresponding timing information and include 1 primary admitting diagnosis code that reflects the principle reason for hospitalization. Each procedure code has corresponding timing information indicating the day of the procedure during the hospitalization. The State Emergency Department Database includes records for hospital-affiliated emergency department visits not resulting in hospitalization. Emergency department visits resulting in hospitalization are included in the SID. The Washington University Human Research Protection Office granted this study an exemption from institutional review board oversight due to the deidentified nature of the data set.

### Study Population

Adult patients (aged ≥18 years) with inpatient records coded for PE and/or DVT were identified from California (2006-2010), Florida (2006-2012), and New York (2006-2011). The earliest hospitalization of a patient with PE or DVT was defined as the index hospitalization and was required to have the preceding 12-month period free of inpatient records coded for PE, DVT, or IVC filter insertion. Contraindications to anticoagulation were identified by *ICD-9-CM* diagnosis or procedure codes and included any of the following: intracranial bleeding, other major bleeding, thrombocytopenia, active gastrointestinal bleeding, aortic dissection, pericardial disease, bacterial endocarditis, threatened abortion, preeclampsia and eclampsia, malignant hypertension, brain surgery, spinal surgery or spinal puncture, and eye surgery coded at the index hospitalization or within the prior 15 days (Table 1). In addition, hemophilia, von Willebrand disease, and cerebral aneurysm coded at the index hospitalization or within the prior year were considered contraindications to anticoagulation. Index hospitalizations with missing sex, residence outside the hospital state, and hospitalization length of stay of more than 6 months were excluded.

**Table 1.** *ICD-9-CM* Diagnosis and Procedure Codes Used to Identify Study Conditions and Procedures

Condition or Procedure	*ICD-9-CM* Codes
Diagnosis	
Deep vein thrombosis	45111, 45119, 4512, 45181, 4519, 4533, 45340, 45341, 45342, 4539, 6713x, 6714x, 6719x
Pulmonary embolism	41511, 41512, 41519, 6346x, 6356x, 6366x, 6376x, 6386, 6396, 6732x, 6733x
Intracranial bleeding	430, 431, 432x, 852x, 853x
Other major bleeding	4560, 45620, 5307, 53082, 53100, 53101, 53120, 53121, 53140, 53141, 53160, 53161, 53200, 53201, 53220, 53221, 53240, 53241, 53260, 53261, 53300, 53301, 53320, 53321, 53340, 53341, 53360, 53361, 53400, 53401, 53420, 53421, 53440, 53441, 53460, 53461, 5693, 5780, 5781, 5789, 64000, 64001, 64003, 64080, 64081, 64083, 64090, 64091, 64093, 64100, 64101, 64103, 64110, 64111, 64113, 64120, 64121, 64123, 64130, 64131, 64133, 64180, 64181, 64183, 64190, 64191, 64193, 2463, 2528, 2800, 2851, 2878, 2879, 36361, 36362, 37481, 37632, 37742, 37923, 4590, 53021, 53501, 53511, 53521, 53531, 53541, 53551, 53561, 53571, 56881, 56985, 6021, 6262, 63410, 63411, 63412, 63510, 63511, 63512, 63610, 63611, 63612, 63710, 63711, 63712, 6381, 6391, 66454, 66570, 66571, 66572, 66574, 66600, 66602, 66604, 66610, 66612, 66614, 66620, 66622, 66624, 67430, 67432, 67434, 7725, 7847, 7848, 7863, 9582, 99811, 99812
Active gastrointestinal ulcer	5311x, 5313x, 5315x, 5321x, 5323x, 5325x, 5331x, 5333x, 5335x, 5341x, 5343x, 5345
Hemophilia and von Willebrand disease	2860, 2861, 2862, 2863, 2864
Thrombocytopenia	2873x, 2874, 28984, 2875
Cerebral aneurysm	09487, 4373, 74781
Aortic dissection	4410x
Pericarditis and pericardial effusion	03641, 07421, 09381, 09883, 3910, 393, 420x, 423x
Bacterial endocarditis	03642, 0932x, 09884, 421x
Threatened abortion	6400x
Preeclampsia and eclampsia	6424x, 6425x, 6426x, 6427x
Malignant hypertension	4010, 4020x, 4030x, 4040x, 4050x
Burns	9065, 9066, 9067, 9068, 9069, 9400, 9401, 9402, 9403, 9404, 9405, 9409, 94100, 94101, 94102, 94103, 94104, 94105, 94106, 94107, 94108, 94109, 94110, 94111, 94112, 94113, 94114, 94115, 94116, 94117, 94118, 94119, 94120, 94121, 94122, 94123, 94124, 94125, 94126, 94127, 94128, 94129, 94130, 94131, 94132, 94133, 94134, 94135, 94136, 94137, 94138, 94139, 94140, 94141, 94142, 94143, 94144, 94145, 94146, 94147, 94148, 94149, 94150, 94151, 94152, 94153, 94154, 94155, 94156, 94157, 94158, 94159, 94200, 94201, 94202, 94203, 94204, 94205, 94209, 94210, 94211, 94212, 94213, 94214, 94215, 94219, 94220, 94221, 94222, 94223, 94224, 94225, 94229, 94230, 94231, 94232, 94233, 94234, 94235, 94239, 94240, 94241, 94242, 94243, 94244, 94245, 94249, 94250, 94251, 94252, 94253, 94254, 94255, 94259, 94300, 94301, 94302, 94303, 94304, 94305, 94306, 94309, 94310, 94311, 94312, 94313, 94314, 94315, 94316, 94319, 94320, 94321, 94322, 94323, 94324, 94325, 94326, 94329, 94330, 94331, 94332, 94333, 94334, 94335, 94336, 94339, 94340, 94341, 94342, 94343, 94344, 94345, 94346, 94349, 94350, 94351, 94352, 94353, 94354, 94355, 94356, 94359, 94400, 94401, 94402, 94403, 94404, 94405, 94406, 94407, 94408, 94410, 94411, 94412, 94413, 94414, 94415, 94416, 94417, 94418, 94420, 94421, 94422, 94423, 94424, 94425, 94426, 94427, 94428, 94430, 94431, 94432, 94433, 94434, 94435, 94436, 94437, 94438, 94440, 94441, 94442, 94443, 94444, 94445, 94446, 94447, 94448, 94450, 94451, 94452, 94453, 94454, 94455, 94456, 94457, 94458, 94500, 94501, 94502, 94503, 94504, 94505, 94506, 94509, 94510, 94511, 94512, 94513, 94514, 94515, 94516, 94519, 94520, 94521, 94522, 94523, 94524, 94525, 94526, 94529, 94530, 94531, 94532, 94533, 94534, 94535, 94536, 94539, 94540, 94541, 94542, 94543, 94544, 94545, 94546, 94549, 94550, 94551, 94552, 94553, 94554, 94555, 94556, 94559, 9460, 9461, 9462, 9463, 9464, 9465, 9470, 9471, 9472, 9473, 9474, 9478, 9479, 94800, 94810, 94811, 94820, 94821, 94822, 94830, 94831, 94832, 94833, 94840, 94841, 94842, 94843, 94844, 94850, 94851, 94852, 94853, 94854, 94855, 94860, 94861, 94862, 94863, 94864, 94865, 94866, 94870, 94871, 94872, 94873, 94874, 94875, 94876, 94877, 94880, 94881, 94882, 94883, 94884, 94885, 94886, 94887, 94888, 94890, 94891, 94892, 94893, 94894, 94895, 94896, 94897, 94898, 94899, 9490, 9491, 9492, 9493, 9494, 9495
Trauma	800-839, 850-904, 925-939, 950-957, 990-994
Surgical procedure	
Brain surgery	0112, 0114, 0120, 0121, 0122, 0123, 0124, 0125, 0128, 0129, 0131, 0132, 0139, 0141, 0142, 0151, 0152, 0153, 0159, 0211, 0212, 0213, 0214, 0291, 0292, 0293, 0751, 0752, 0753, 0754, 0759, 0761, 0762, 0763, 0764, 0765, 0768, 0769, 0771, 0772, 0779, 3801, 3811, 3831, 3841, 3851, 3861, 3881, 3928
Spine surgery	810x, 813x, 0301, 0302, 0309, 8050, 8051, 8053, 8054, 8059, 8460, 8461, 8462, 8463, 8464, 8465, 8466, 8467, 8468, 8469, 8480, 8481, 8482, 8483, 8484, 8485, 8161, 8162, 8163, 8164, 8451, 0332, 0339, 034
Eye surgery	1160, 1161, 1162, 1163, 1164, 1169, 1251, 1252, 1253, 1254, 1255, 1259, 1261, 1262, 1263, 1264, 1265, 1266, 1267, 1269, 1271, 1272, 1273, 1274, 1279, 1311, 1319, 132, 133, 1341, 1342, 1343, 1351, 1359, 1361, 1362, 1363, 1364, 1365, 1366, 1369, 1370, 1371, 1372, 138, 139, 1390, 1391, 1431, 1432, 1433, 1434, 1435, 1439, 1441, 1449, 1451, 1452, 1453, 1454, 1455, 1459, 1421, 1422, 1423, 1424, 1425, 1426, 1427, 1429, 1200, 1201, 1202, 1211, 1212, 1213, 1214, 1231, 1232, 1233, 1234, 1235, 1239, 1240, 1241, 1242, 1243, 1244, 1281, 1282, 1283, 1284, 1285, 1286, 1287, 1288, 1289, 1291, 1292, 1293, 1297, 1298, 1299, 1300, 1301, 1302, 1400, 1401, 1402, 146, 1471, 1472, 1473, 1474, 1475, 1479, 1481, 1482, 1483, 149
Spinal puncture	0331, 8721
Embolectomy and thrombectomy or endarterectomy of thoracic vessels (other than aorta)	3805, 3815
Hip replacement	0070, 0071, 0072, 0073, 0085, 0086, 0087, 8151, 8152, 8153
Knee replacement	0080, 0081, 0082, 0083, 0084, 8154, 8155

Abbreviation: *ICD-9-CM*, *International Classification of Diseases, Ninth Revision, Clinical Modification*.

### Primary Exposure, Outcomes, and Other Baseline Characteristics

During the index hospitalization, IVC filter insertion was identified by the *ICD-9-CM* procedure code 38.7. Comorbidities were identified using the Elixhauser classification^16^ derived from the index hospitalization and admissions within the preceding year. Medical insurance (Medicare, Medicaid, private insurance, and other) and admission through the emergency department were identified at the index hospitalization.

### Statistical Analysis

Our primary method of analysis was a multivariable Cox model with IVC filter status as a time-dependent variable to account for immortal time bias. The start time for this analysis was the date of index hospitalization. Patients were followed up until the time of an event or censored at 30 days. Patients with IVC filters were not identified until the time of procedure to allow for a time-dependent IVC filter status. The following variables were used in the multivariable, time-dependent Cox model and to build a propensity score with a logistic regression model: age, sex, primary payer, admission through emergency department, thromboembolism, intracranial bleeding, other major bleeding, thrombocytopenia, active gastrointestinal ulcer, hemophilia or von Willebrand disease, cerebral aneurysm, aortic dissection, pericardial disease, bacterial endocarditis, preeclampsia and eclampsia, malignant hypertension, brain surgery, spinal surgery, eye surgery, congestive heart failure, valvular disease, pulmonary circulation disease, peripheral vascular disease, paralysis, other neurologic disorders, chronic obstructive pulmonary disease, diabetes, hypertension, hypothyroidism, renal failure, liver disease, peptic ulcer disease, lymphoma, metastatic cancer, solid tumors without metastasis, rheumatoid arthritis, coagulopathy, obesity, weight loss, fluid and electrolyte disorders, chronic blood loss anemia, alcohol abuse, drug abuse, psychoses, and depression. A second Cox model was created that included the original variables and the propensity score as an additional adjustment variable. The follow-up period started at the admission date of the index hospitalization for individuals with and without IVC filter insertion. The primary outcome of interest was mortality at 30 days. Baseline characteristics of patients with and without IVC filter were compared using 2-sample *t* test for continuous variables and χ^2^ test for categorical data. To account for any overdispersion, the Pearson χ^2^ test was used to adjust standard errors via quasi-likelihood estimation. A 2-sided *t* test was used, and *P* < .05 was considered statistically significant. All analyses were conducted using SAS, version 9.3 and SAS Enterprise Guide, version 7.1 (SAS Institute Inc).

## Results

We identified 132 355 patients 18 years and older with an *ICD-9-CM* hospitalization code for PE, DVT, or both along with a contraindication to anticoagulation from California (2006-2010), Florida (2006-2012), and New York (2006-2011). After application of exclusion criteria, 126 124 patients remained in the study. Of these, 94 patients with IVC filter had incomplete data for analysis, leaving 126 030 patients in the final study population (Figure), with 45 771 (36.3%) receiving an IVC filter and 80 259 (63.7%) who did not receive an IVC filter. Baseline characteristics and coexisting conditions are presented in Table 2. Of the 126 030 patients, 61 281 (48.6%) were male and the mean (SD) age was 66.9 (16.6) years. The median time to IVC filter placement was 5.0 days (interquartile range, 2.0-11.0 days).

**Figure.**
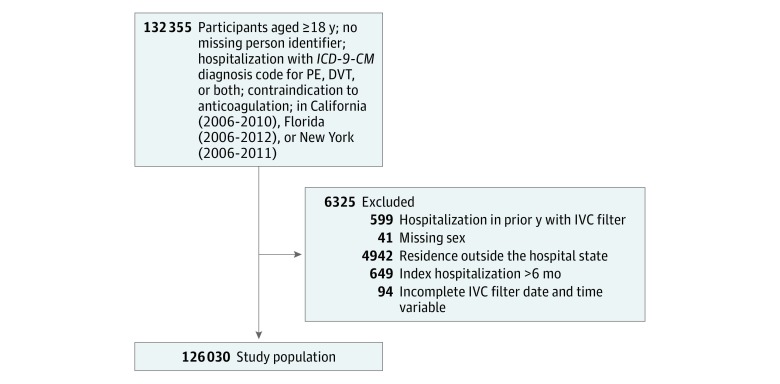
Derivation of the Study Population DVT indicates deep venous thrombosis; *ICD-9-CM*, *International Classification of Diseases, Ninth Revision, Clinical Modification;* IVC, inferior vena cava; PE, pulmonary embolism.

**Table 2.** Patient Characteristics

Characteristics	No. (%)		
	Overall (n = 126 030)	No IVC Filter (n = 80 259)	IVC Filter (n = 45 771)
Male	61 281 (48.6)	38 673 (48.2)	22 608 (49.4)
Age at admission, mean (SD), y	66.9 (16.6)	65.7 (17.1)	69.1 (15.6)
Primary expected payer			
Medicare	76 383 (60.6)	47 036 (58.6)	29 347 (64.1)
Medicaid	12 654 (10.0)	8500 (10.6)	4154 (9.1)
Private insurance	29 011 (23.0)	19 070 (23.8)	9941 (21.7)
Self-pay	3289 (2.6)	2405 (3.0)	884 (1.9)
No charge	743 (0.6)	548 (0.7)	195 (0.4)
Other	3950 (3.1)	2700 (3.4)	1250 (2.7)
Payment method			
Government insurance	89 037 (70.6)	55 536 (69.2)	33 501 (73.2)
Private insurance	29 011 (23.0)	19 070 (23.8)	9941 (21.7)
Other	7982 (6.3)	5653 (7.0)	2329 (5.1)
ED visit	99 818 (79.2)	62 770 (78.2)	37 048 (80.9)
Thromboembolism			
PE and DVT	19 271 (15.3)	9263 (11.5)	10 008 (21.9)
PE only	42 398 (33.6)	32 447 (40.4)	9951 (21.7)
DVT only	64 361 (51.1)	38 549 (48.0)	25 812 (56.4)
Intracranial bleeding	9691 (7.7)	3574 (4.5)	6117 (13.4)
Other major bleeding	71 455 (56.7)	43 216 (53.8)	28 239 (61.7)
Thrombocytopenia	37 624 (29.9)	25 743 (32.1)	11 881 (26.0)
Active gastrointestinal ulcer	704 (0.6)	434 (0.5)	270 (0.6)
Hemophilia or von Villebrand disease	374 (0.3)	210 (0.3)	164 (0.4)
Cerebral aneurysm	448 (0.4)	197 (0.2)	251 (0.5)
Aortic dissection	833 (0.7)	575 (0.7)	258 (0.6)
Pericardial disease	6121 (4.9)	4583 (5.7)	1538 (3.4)
Endocarditis	3857 (3.1)	3448 (4.3)	409 (0.9)
Preeclampsia and eclampsia	111 (0.1)	96 (0.1)	15 (0.0)
Malignant hypertension	3342 (2.7)	2468 (3.1)	874 (1.9)
Cranial surgery	5124 (4.1)	1723 (2.1)	3401 (7.4)
Spinal surgery	6331 (5.0)	3651 (4.5)	2680 (5.9)
Recent lumbar puncture	5224 (4.1)	3628 (4.5)	1596 (3.5)
Eye surgery	91 (0.1)	73 (0.1)	18 (0.0)
Congestive heart failure	33 850 (26.9)	21 612 (26.9)	12 238 (26.7)
Valvular disease	17 974 (14.3)	11 901 (14.8)	6073 (13.3)
Diseases of pulmonary vasculature	65 822 (52.2)	44 343 (55.2)	21 479 (46.9)
Peripheral vascular disease	17 954 (14.2)	11 576 (14.4)	6378 (13.9)
Paralysis	12 998 (10.3)	6522 (8.1)	6466 (14.1)
Other neurological disorders	23 289 (18.5)	12 978 (16.2)	10 311 (22.5)
Chronic obstructive pulmonary disease	37 618 (29.8)	24 210 (30.2)	13 408 (29.3)
Diabetes	37 830 (30.0)	23 617 (29.4)	14 213 (31.1)
Hypertension	87 354 (69.3)	54 783 (68.3)	32 571 (71.2)
Hypothyroidism	17 839 (14.2)	11 245 (14.0)	6594 (14.4)
Renal failure	28 916 (22.9)	18 355 (22.9)	10 561 (23.1)
Liver disease	7932 (6.3)	5136 (6.4)	2796 (6.1)
Peptic ulcer disease	67 625 (53.7)	42 733 (53.2)	24 892 (54.4)
Lymphoma	3788 (3.0)	2372 (3.0)	1416 (3.1)
Metastatic cancer	17 018 (13.5)	9777 (12.2)	7241 (15.8)
Solid tumor without metastasis	24 647 (19.6)	14 035 (17.5)	10 612 (23.2)
Rheumatoid arthritis and/or collagen vascular disease	5854 (4.6)	3824 (4.8)	2030 (4.4)
Coagulopathy	44 212 (35.1)	29 535 (36.8)	14 677 (32.1)
Obesity	20 793 (16.5)	13 631 (17.0)	7162 (15.6)
Recent weight loss	22 412 (17.8)	13 223 (16.5)	9189 (20.1)
Fluid and electrolyte disorders	70 232 (55.7)	42 924 (53.5)	27 308 (59.7)
Chronic blood loss anemia	15 152 (12.0)	8671 (10.8)	6481 (14.2)
Deficiency anemias	57 670 (45.8)	36 132 (45.0)	21 538 (47.1)
Alcohol abuse	8039 (6.4)	5089 (6.3)	2950 (6.4)
Drug abuse	5889 (4.7)	4465 (5.6)	1424 (3.1)
Psychoses	8950 (7.1)	5614 (7.0)	3336 (7.3)
Depression	19 382 (15.4)	12 312 (15.3)	7070 (15.4)

Abbreviations: DVT, deep venous thrombosis; ED, emergency department; IVC, inferior vena cava; PE, pulmonary embolism.

When evaluated in a multivariable Cox model with IVC filter placement analyzed as a time-dependent variable to account for immortal time bias, IVC filter placement was associated with an increased hazard ratio of mortality (1.18; 95% CI, 1.13-1.22; *P* < .001). After the addition of the propensity score to the multivariable Cox model, IVC filter placement continued to be associated with an increased hazard ratio of mortality (1.18; 95% CI, 1.13-1.22; *P* < .001).

## Discussion

The most significant finding of this study of IVC filter use in patients with VTE and a contraindication to anticoagulation is that treatment with an IVC filter was associated with a higher 30-day mortality than treatment without IVC filter placement after adjustment for demographics, comorbidities, immortal times bias, and the propensity to receive a filter.

The initial long-term evaluation of IVC filter use was by Greenfield et al in 1981^17^ and was expanded in the late 1980s^18^ to include 469 patients. This study showed a 4% rate of PE in patients after placement of an IVC filter but had suboptimal follow-up and lacked a control group. Largely based on this work, IVC filter implantation increased from 2000 procedures in 1979 to more than 100 000 in 2005.^9^ At present, there are 2 randomized clinical trials evaluating the long-term outcomes of IVC filter use. The first, originally published in 1998^11^ with a follow-up report in 2005,^19^ found that patients with VTE who were randomized to receive IVC filters experienced a reduction in symptomatic PE, no change in mortality, and an increased risk of recurrent DVT. Importantly, this trial excluded patients with a contraindication to anticoagulation, negating its applicability to the subset of patients in whom IVC filters are most universally recommended. The second study,^12^ published in 2015, randomized 399 patients with PE and a high probability of recurrence to anticoagulation and a retrievable IVC filter vs anticoagulation alone. The outcomes of the 2 groups did not differ with respect to the primary outcome of recurrent PE or secondary outcomes including death or DVT at 3 or 6 months. Because both groups had anticoagulation, patients with a contraindication to anticoagulation were excluded. In contrast, our study evaluates a diverse patient population with multiple contraindications to anticoagulation and not only extends the findings of prior IVC filter publications but also adjusts for the effect of immortal time in observational studies to bias results in favor of intervention.

Given the widespread use of IVC filters and persistent questions regarding their efficacy, several observational studies within the last 5 years have attempted to further evaluate the association of IVC filter use and mortality.^2,20,21,22^ These studies, which involved different subgroups of patients with VTE having varying ability to tolerate anticoagulation, were similar in that none of them adjusted for immortal time bias. In contrast, a 2016 study^23^ did attempt to address the issue of immortal time bias in the assessment of IVC filter efficacy. This study retrospectively analyzed patients without cancer in California with acute VTE and included both patients who could tolerate anticoagulation and those with a contraindication. The only group of patients in whom IVC filter use significantly reduced the short-term risk of death was the approximately 3000-patient subset with acute VTE and a contraindication to anticoagulation owing to active bleeding. Referring an actively bleeding patient for an invasive procedure is likely to involve selection bias whereby the sickest patients are considered too high risk for the procedure and are relegated to the control group, thus possibly confounding the results. This study attempts to expand on these findings by analyzing a diverse patient cohort including multiple states, patients with cancer, patients with a contraindication to anticoagulation, and patients who are and are not actively bleeding.

### Limitations

Our findings should be interpreted within the context of several limitations. This study is retrospective and uses observational data derived from codes designed for reimbursement. A 2016 study proposed that the use of diagnostic codes from claims data can lead to an underestimation of event rates.^24^ Furthermore, retrospective observational studies may be subject to various types of bias that persist despite various techniques to adjust for differences in baseline characteristics. Therefore, these results should be considered hypothesis generating only. Second, this study only captured patient deaths during the index hospitalization, on a repeated admission or at a subsequent emergency department visit in the same state as the index hospitalization, thereby allowing for the possibility that some out-of-hospital or out-of-state deaths were not captured. However, there is no reason to believe these uncaptured events would occur more frequently in one group than in the other. Third, contraindications to anticoagulation span a range from minor relative contraindications to severe absolute contraindications. The lack of granularity of administrative data precludes the determination of the degree of absoluteness of any patient’s contraindication to anticoagulation, whether therapeutic anticoagulation was attempted but required discontinuation, or if a patient was a suitable candidate for an IVC filter. Fourth, we are unable to determine whether the IVC filters used were retrievable and whether they were ever retrieved.

## Conclusions

This analysis suggests that patients with a contraindication to anticoagulation who receive an IVC filter have an increased risk of death at 30 days after adjustment for baseline differences, comorbidities, immortal time bias, and propensity score compared with similar patients who did not receive an IVC filter. Randomized clinical trials are required to determine the efficacy of IVC filter placement in patients with VTE and a contraindication to anticoagulation.

## References

[1] Heit JA The epidemiology of venous thromboembolism in the community: implications for prevention and management. J Thromb Thrombolysis. 2006;21(1):-. 16475038 10.1007/s11239-006-5572-y

[2] Bikdeli B, Wang Y, Minges KE, Vena caval filter utilization and outcomes in pulmonary embolism: Medicare hospitalizations from 1999 to 2010. J Am Coll Cardiol. 2016;67(9):1027-1035. 26940921 10.1016/j.jacc.2015.12.028 PMC5322943

[3] Kearon C, Akl EA, Comerota AJ, Antithrombotic therapy for VTE disease: antithrombotic therapy and prevention of thrombosis, 9th ed: American College of Chest Physicians evidence-based clinical practice guidelines. Chest. 2012;141(suppl 2):e419S-e496S. 22315268 10.1378/chest.11-2301 PMC3278049

[4] Jaff MR, McMurtry MS, Archer SL, ; American Heart Association Council on Cardiopulmonary, Critical Care, Perioperative and Resuscitation; American Heart Association Council on Peripheral Vascular Disease; American Heart Association Council on Arteriosclerosis, Thrombosis and Vascular Biology Management of massive and submassive pulmonary embolism, iliofemoral deep vein thrombosis, and chronic thromboembolic pulmonary hypertension: a scientific statement from the American Heart Association. Circulation. 2011;123(16):1788-1830. 21422387 10.1161/CIR.0b013e318214914f

[5] Kaufman JA, Kinney TB, Streiff MB, Guidelines for the use of retrievable and convertible vena cava filters: report from the Society of Interventional Radiology multidisciplinary consensus conference. J Vasc Interv Radiol. 2006;17(3):449-459. 16567669 10.1097/01.rvi.0000203418-39769.0d

[6] Caplin DM, Nikolic B, Kalva SP, Ganguli S, Saad WE, Zuckerman DA; Society of Interventional Radiology Standards of Practice Committee Quality improvement guidelines for the performance of inferior vena cava filter placement for the prevention of pulmonary embolism. J Vasc Interv Radiol. 2011;22(11):1499-1506. 21890380 10.1016/j.jvir.2011.07.012

[7] Kinney TB, Aryafar H, Ray CE Jr, ; American College of Radiology Expert Panel on Interventional Radiology ACR Appropriateness Criteria: Radiologic Management of Inferior Vena Cava Filters. Reston, VA: American College of Radiology; 2012.

[8] Baglin TP, Brush J, Streiff M; British Committee for Standards in Haematology Writing Group Guidelines on use of vena cava filters. Br J Haematol. 2006;134(6):590-595. 16869824 10.1111/j.1365-2141.2006.06226.x

[9] Moore PS, Andrews JS, Craven TE, Trends in vena caval interruption. J Vasc Surg. 2010;52(1):118-125.e3, discussion 125-126. 20304583 10.1016/j.jvs.2009.09.067

[10] Saeed MJ, Turner TE, Brown DL Trends in inferior vena cava filter placement by indication in the United States from 2005 to 2014. JAMA Intern Med. 2017;177(12):1861-1862. 29114739 10.1001/jamainternmed.2017.5960 PMC5820719

[11] Decousus H, Leizorovicz A, Parent F, ; Prévention du Risque d’embolie Pulmonaire par Interruption Cave Study Group A clinical trial of vena caval filters in the prevention of pulmonary embolism in patients with proximal deep-vein thrombosis. N Engl J Med. 1998;338(7):409-415. 9459643 10.1056/NEJM199802123380701

[12] Mismetti P, Laporte S, Pellerin O, ; Effect of a retrievable inferior vena cava filter plus anticoagulation vs anticoagulation alone on risk of recurrent pulmonary embolism: a randomized clinical trial. JAMA. 2015;313(16):1627-1635. 25919526 10.1001/jama.2015.3780

[13] Lévesque LE, Hanley JA, Kezouh A, Suissa S Problem of immortal time bias in cohort studies: example using statins for preventing progression of diabetes. BMJ. 2010;340:b5087. 20228141 10.1136/bmj.b5087

[14] Healthcare Cost and Utilization Project, Agency for Healthcare Research and Quality Healthcare Cost and Utilization Project state inpatient databases, 2006-2012. http://www.hcup-us.ahrq.gov/sidoverview.jsp. Accessed May 8, 2015. 21413206

[15] Healthcare Cost and Utilization Project, Agency for Healthcare Research and Quality Healthcare Cost and Utilization Project state emergency department databases, 2006-2012. http://www.hcup-us.ahrq.gov/seddoverview.jsp. Accessed May 8, 2015. 21413206

[16] Elixhauser A, Steiner C, Harris DR, Coffey RM Comorbidity measures for use with administrative data. Med Care. 1998;36(1):8-27. 9431328 10.1097/00005650-199801000-00004

[17] Greenfield LJ, Peyton R, Crute S, Barnes R Greenfield vena caval filter experience: late results in 156 patients. Arch Surg. 1981;116(11):1451-1456. 7305658 10.1001/archsurg.1981.01380230065010

[18] Greenfield LJ, Michna BA Twelve-year clinical experience with the Greenfield vena caval filter. Surgery. 1988;104(4):706-712. 3175867

[19] PREPIC Study Group Eight-year follow-up of patients with permanent vena cava filters in the prevention of pulmonary embolism: the PREPIC (Prevention du Risque d’Embolie Pulmonaire par Interruption Cave) randomized study. Circulation. 2005;112(3):416-422. 16009794 10.1161/CIRCULATIONAHA.104.512834

[20] Muriel A, Jiménez D, Aujesky D, ; RIETE Investigators Survival effects of inferior vena cava filter in patients with acute symptomatic venous thromboembolism and a significant bleeding risk. J Am Coll Cardiol. 2014;63(16):1675-1683. 24576432 10.1016/j.jacc.2014.01.058

[21] Stein PD, Matta F Vena cava filters in unstable elderly patients with acute pulmonary embolism. Am J Med. 2014;127(3):222-225. 24280176 10.1016/j.amjmed.2013.11.003

[22] Isogai T, Yasunaga H, Matsui H, Tanaka H, Horiguchi H, Fushimi K Effectiveness of inferior vena cava filters on mortality as an adjuvant to antithrombotic therapy. Am J Med. 128(3):312.e23-312.e31. 25446296 10.1016/j.amjmed.2014.10.034

[23] White RH, Brunson A, Romano PS, Li Z, Wun T Outcomes after vena cava filter use in noncancer patients with acute venous thromboembolism: a population-based study. Circulation. 2016;133(21):2018-2029. 27048765 10.1161/CIRCULATIONAHA.115.020338

[24] Psaty BM, Delaney JA, Arnold AM, study of cardiovascular health outcomes in the era of claims data: the cardiovascular health study. Circulation. 2016;133(2):156-164. doi:10.1161/CIRCULATIONAHA.115.018610 26538580 PMC4814341

